# A Measure of Community Exposure: PFOA in Well Water Correlates with Serum Levels

**DOI:** 10.1289/ehp.119-a35a

**Published:** 2011-01

**Authors:** Kellyn S. Betts

**Affiliations:** **Kellyn S. Betts** has written about environmental contaminants, hazards, and technology for solving environmental problems for publications including *EHP* and *Environmental Science & Technology* for more than a dozen years

The first detailed investigation into contamination of private wells with perfluorooctanoic acid (PFOA) and levels of the compound in human blood serum suggests that drinking water was the dominant source of exposure to PFOA in a community industrially exposed to the compound **[*EHP*** **119(1):92–97; Hoffman et al.]**. The study, conducted in 2005 and 2006, included only people who obtained their drinking water from private wells. The results showed that each 1-μg/L increase of the compound in the participants’ water supply was associated with a 141.5-μg/L increase in people’s serum PFOA concentrations.

The participants lived around DuPont’s Washington Works facility in Parkersburg, West Virginia, where PFOA (also known as C8) is used in the manufacture of Teflon nonstick polymers. PFOA has been shown to increase risk of cancer, reproductive problems, and liver damage in laboratory animals, although human health effects are less clear. Many of the water monitoring data used in this study were collected as part of an agreement between DuPont and the U.S. Environmental Protection Agency (EPA) to conduct a human health risk assessment for PFOA.

The groundwater in the Parkersburg area had been contaminated by DuPont’s releases of PFOA into the nearby Ohio River. A second source of contamination was PFOA that was released into the atmosphere and deposited onto soils, which then leached into the groundwater.

Previous research in this study area linked drinking water supplied by six local water districts and consumption of home-grown vegetables to PFOA levels in participants’ serum [*EHP* 118(8):1100–1108; Steenland et al.]. The new study provides a quantitative estimate of the relationship between drinking water and serum PFOA levels based on exposure to a wider range of PFOA levels in drinking water from 62 wells. It also corroborates the earlier finding about consumption of home-grown vegetables.

Many of the wells in the study had PFOA concentrations that exceeded the EPA’s 0.4-μg/L advisory level, although the median concentration in the well water samples was half that level. The concentrations of PFOA in participants’ serum ranged from 0.9 to 4,751 μg/L, with a median of 75.7 μg/L, approximately 20 times the average level in the U.S. general population.

The association between PFOA in drinking water and serum was similar for both shorter- and longer-term residents of the area. The researchers found the associations held after excluding participants who reported drinking bottled water and those who worked at the DuPont facility. Compared with other factors (including age, sex, body weight, cigarette smoking, and alcohol consumption), drinking water was consistently the strongest predictor of serum PFOA levels.

The 141.5:1 ratio estimated for drinking water to serum PFOA concentrations is close to the 114:1 ratio predicted by a steady-state pharmacokinetic model employed by the authors. These findings may be useful in developing drinking water guidelines and studying other communities where PFOA is manufactured.

